# Identification of risk factors and construction of a prediction model for multidrug-resistant organism infections in neutropenic patients

**DOI:** 10.3389/fcimb.2026.1805777

**Published:** 2026-04-15

**Authors:** Dian Jin, Qianqian Yang, Wenxiu Shu, Jing Le, Bingrong Chen

**Affiliations:** Department of Hematology, Ningbo Medical Center Lihuili Hospital, Ningbo, China

**Keywords:** infection, multidrug-resistant organism, neutropenia, prediction model, risk factors

## Abstract

**Objective:**

Multidrug-resistant organisms (MDROs) pose a severe threat to neutropenic patients with compromised immunity, leading to poor outcomes and increased healthcare burdens. This study aimed to develop and validate a prediction model for MDRO infections in this population.

**Methods:**

A total of 391 neutropenic patients (206 in training cohort, 185 in validation cohort) admitted to Ningbo Medical Center Lihuili Hospital, from January 2023 to December 2024 were enrolled. Demographic, clinical, and outcome data were collected. The least absolute shrinkage and selection operator (LASSO) regression and multivariate logistic regression analyses were used to identify independent risk factors, and a nomogram was constructed for prediction. Model performance was evaluated via receiver operating characteristic (ROC),calibration curves and decision curve analysis (DCA).

**Results:**

MDRO infection occurred in 14.1% of patients, associated with longer hospital stays, higher costs, and increased mortality. Independent risk factors included comorbid cardiac disease (OR = 13.500, 95%CI: 2.484-73.384, P = 0.003), ECOG score≥2 (OR = 3.210, 95%CI: 1.114-9.255, P = 0.031), neutropenia duration≥7 days (OR = 4.028, 95%CI: 1.399-11.600, P = 0.010), and broad-spectrum antibiotic use in 3 months prior (OR = 13.053, 95%CI: 2.419-70.441, P = 0.003). The nomogram demonstrated good discriminative ability, with an area under the ROC curve of 0.874 in the training cohort and 0.764 in the validation cohort. Calibration curves confirmed favorable prediction accuracy and the DCA showed good clinical applicability.

**Conclusions:**

This study identifies key risk factors and provides a practical predictive tool for early identification of high-risk patients, enabling targeted interventions to reduce MDRO infections, improve patient outcomes, and alleviate healthcare burdens.

## 1 Introduction

Multidrug-resistant organisms (MDROs) typically refer to bacteria resistant to three or more classes of antibiotics (Magiorakos et al., 2012). They primarily include methicillin-resistant Staphylococcus aureus (MRSA), vancomycin-resistant Enterococci (VRE), extended-spectrum β-lactamase (ESBL)-producing Enterobacteriaceae, carbapenem-resistant Enterobacteriaceae (CRE), carbapenem-resistant Acinetobacter baumannii (CRAB), Multidrug-resistant/extensively drug-resistant Pseudomonas aeruginosa (MDR/PDR-PA) and carbapenem-resistant Pseudomonas aeruginosa (CRPA). Compared to antibiotic-sensitive microorganisms, infections caused by MDROs are more difficult to control, with significantly higher mortality rates, longer hospital stays, and increased healthcare costs (Serra-Burriel et al., 2020; Gussin et al., 2024). Currently, antibiotic resistance poses a major threat to public health (Laxminarayan, 2022).

Neutropenia is a common complication in cancer patients, particularly those with hematologic malignancies undergoing chemotherapy or hematopoietic stem cell transplantation (HSCT), as it significantly increases the risk of infection due to immunodeficiency (Kuderer et al., 2006; Stohs et al., 2024). More than 80% of patients with hematologic malignancies and 10%-50% of those with solid tumors develop neutropenia-related infections after at least one cycle of chemotherapy (Klastersky, 2004; Freifeld et al., 2011; Stohs et al., 2024). Neutropenic infections are associated with high mortality, with bloodstream infection-related mortality rates ranging from 7.1% to 42% (Yan et al., 2018; Chinese Society of Hematology CMA and Chinese Medical Doctor Association HB, 2020). Neutropenia has been recognized as a well-established predictor of severe infection risk and infection-related death in cancer patients (Sickles et al., 1975).

Neutropenic patients, due to their immunocompromised state and prolonged hospital stays, are frequently subjected to long-term prophylactic and therapeutic use of broad-spectrum antibiotics, which can disrupt microbial balance and significantly increase the risk of MDRO infections (Averbuch et al., 2013). MDRO infections in neutropenic patients can lead to severe outcomes. Reported mortality rates for acute leukemia patients with neutropenia and MDRO infections reach as high as 25%-30%, significantly exceeding those caused by antibiotic-sensitive microorganisms (Scheich et al., 2018).

Data from China indicate that in recent years, the incidence of bloodstream infections caused by ESBL-producing Escherichia coli, ESBL-producing Klebsiella pneumoniae, carbapenem-resistant Klebsiella pneumoniae, CRPA, and CRAB in neutropenic patients ranged from 39.1%-68.3%, 7.3%-41.2%, 0.5%-11.4%, 0%-3.2%, and 5.7%-7.8%, respectively (Chinese Society of Hematology CMA and Chinese Medical Doctor Association HB, 2020). Moreover, the prevalence of MDRO infections has shown an increasing trend year by year. Therefore, it is imperative to take proactive and effective measures to reduce the occurrence and transmission of MDRO infections in neutropenic patients.

Based on the above clinical challenges, we proposed the predefined research hypothesis for this study: neutropenic patients with specific clinical characteristics may have a significantly increased risk of MDRO infections; and a clinical prediction model constructed based on these identified independent risk factors can effectively stratify the MDRO infection risk in this population, providing a practical and actionable tool for clinical infection control and targeted anti-infective interventions. To verify the above hypothesis and address the unmet clinical need of early identification of high-risk patients for MDRO infections, we conducted this hypothesis-driven retrospective cohort study utilizing real-world data from neutropenic patients in our center, with the primary research aim to identify the independent clinical risk factors for MDRO infections in neutropenic patients and to develop a prediction model based on these key risk factors, thereby realizing clinical risk stratification of MDRO infections for neutropenic patients and providing a reliable reference for clinical empirical anti-infective therapy and infection control strategies.

## 2 Materials and methods

### 2.1 Patients

The study included neutropenic patients admitted to the Department of Hematology at Ningbo Medical Center Lihuili Hospital between January 1, 2023, and December 31, 2024. Data for the study were collected by reviewing patients’ electronic medical records. The inclusion criteria were as follows (Magiorakos et al., 2012): met the diagnostic criteria for neutropenia (Gussin et al., 2024); hospitalized patients (Serra-Burriel et al., 2020); age≥18 years. The exclusion criteria were as follows (Magiorakos et al., 2012): were suffering from psychosis (Gussin et al., 2024); had incomplete data. Permission to conduct the study was granted by the Ethical Review Committee of Ningbo Medical Center Lihuili Hospital (approval No. KY2025PJ347). Written informed consent was obtained from all patients.

### 2.2 Cohort definition and variable recode

Patients admitted between January 1, 2023, and December 31, 2023, were assigned to the training cohort for variable screening and model development. Patients admitted between January 1, 2024, and December 31, 2024, were assigned to the validation cohort to verify the results obtained from the training cohort. Patient demographics and clinical parameters were collected, including gender, age, duration of neutropenia, albumin level, diagnosis of acute myeloid leukemia (AML)/myelodysplastic syndrome (MDS), comorbidities (hypertension, diabetes, pulmonary comorbidities, cardiac comorbidities), number of hospitalizations, performance status (PS), body mass index (BMI), neutropenic fever episodes within the past 1 year, prior hospitalization in other centers within the past 1 year, antibiotic prophylaxis, antifungal prophylaxis, invasive procedure during current admission, presence of central venous catheter, history of MDRO infection, broad-spectrum antibiotic use within the past 3 months, intensive care unit (ICU) transfer during current admission, and post-HSCT status. Patients were monitored for MDRO infection and infection-related mortality during hospitalization and within 3 months after discharge. Additionally, length of hospital-stay and hospitalization costs were recorded. The hospitalization cost refers to all expenses incurred during the entire hospital stay.

### 2.3 Microbiological identification and infection determination

All clinical isolates were identified using matrix-assisted laser desorption ionization time-of-flight mass spectrometry (MALDI-TOF MS, EXS3000, Zybio, China). Antimicrobial susceptibility testing was performed using the VITEK 2 automated system (bioMérieux, France), with results interpreted according to the Clinical and Laboratory Standards Institute (CLSI) 2023 standards. We clearly specified the infection sites involved in this study, which mainly include respiratory tract infection, bloodstream infection, digestive tract infection, urinary tract infection, and skin and soft tissue infection. Infection was attributed to MDRO if the following two conditions were met (1): MDRO was isolated from a clinically relevant specimen of the infected site (2); the patient presented with corresponding clinical symptoms and signs of infection, and no other pathogenic microorganisms that could explain the infection were found.

### 2.4 Definitions

In this study, we adopted a composite MDRO endpoint encompassing MRSA, VRE, ESBL-producing Gram-negative bacteria, CRE, CRAB, CRPA, MDR-PA, and clostridioides difficile, which is consistent with the internationally recognized MDRO definition (Magiorakos et al., 2012) and the clinical research consensus for neutropenic infections (Averbuch et al., 2013; Scheich et al., 2018). Neutropenia was defined as an absolute neutrophil count < 0.5 × 10^9^/L. Cardiac comorbidities include coronary heart disease, heart valvular disease, arrhythmias, or other diseases that cause heart dysfunction. Pulmonary comorbidities include pulmonary ventilation dysfunction such as chronic obstructive pulmonary disease and diffuse dysfunction diseases such as interstitial lung disease. PS was graded according to the Eastern Cooperative Oncology Group (ECOG) (Oken et al., 1982) as follows: 0, fully active, able to carry on all pre-disease performance without restriction; 1, restricted in physically strenuous activity but ambulatory and able to carry out work of a light or sedentary nature; 2, ambulatory and capable of all self-care but unable to carry out any work activities; up and about more than 50% of waking hours; 3, capable of only limited self-care; confined to bed or chair more than 50% of waking hours; 4, completely disabled; cannot carry on any self-care; totally confined to bed or chair; 5, dead.

### 2.5 Statistical analysis

Statistical analyses were performed using SPSS 26.0 and R 4.0 software. Categorical variables were presented as absolute counts and percentages. Group comparisons were performed using the chi-square test or Fisher’s exact probability test based on sample size and expected frequencies. Continuous variables are described using median and range, and intergroup comparisons are performed using the Mann-Whitney U test. In the training cohort, MDRO infection was set as the dependent variable, while 23 patient-related clinical characteristics served as independent variables. We first performed the least absolute shrinkage and selection operator (LASSO) regression with 10-fold cross-validation to screen for clinically meaningful variables associated with MDRO infection. The LASSO regression was implemented by R software (glmnet package), with the optimal penalty parameter (λ) determined by the lambda.min (the λ value with the minimum binomial deviance) from the cross-validation curve. The eight potential risk factors selected by LASSO regression were included in the multivariate logistic regression to determine independent risk factors associated with MDRO infection and to construct a nomogram score prediction model. Model performance was evaluated in both training and validation cohorts using receiver operating characteristic (ROC) curves and calibration curves. Decision curve analysis (DCA) was used to assess clinically effective. The 95% confidence intervals (CIs) were used to estimate odds ratios (ORs). Statistical significance was defined as P<0.05 for all analyses.

## 3 Results

### 3.1 Characteristics and outcomes of patients

A total of 391 patients with neutropenia were enrolled in this study, with 206 patients assigned to the training cohort and 185 patients assigned to the validation cohort. Twenty-three patient-related clinical characteristics and the final MDRO infection status are presented in **Table 1**. In the overall population, the median age was 59 years (range 18-87), 51.4% were diagnosed with AML or MDS, and 14.1% ultimately developed MDRO infection. The training and validation cohorts were comparable in terms of demographic and clinical characteristics (P>0.05).

**Table 1:** Demographic and clinical characteristics of patients.

Characteristic	Whole population [cases (%)]	Training cohort [cases (%)]	Validation cohort [cases (%)]	*P* value
Total	391	206	185	
Age≥75	39(10.0)	16(7.8)	23(12.4)	0.124
Male	227(58.1)	115(55.8)	112(60.5)	0.345
AML or MDS	201(51.4)	102(49.5)	99(53.5)	0.430
Comorbidities				
Hypertension	107(27.4)	50(24.3)	57(30.8)	0.148
Diabetes	47(12.0)	19(9.2)	28(15.1)	0.073
Pulmonary comorbidity	35(9.0)	20(9.7)	15(8.1)	0.580
Cardiac comorbidity	40(10.2)	17(8.3)	23(12.4)	0.173
ECOG≥2	114(29.2)	62(30.1)	52(28.1)	0.666
BMI				
Obesity(BMI≥30)	8(2.0)	7(3.4)	1(0.5)	0.102
Malnutrition(BMI<18.5)	34(9.7)	20(7.6)	14(8.7)	0.453
Neutropenia lasting ≥7 days	150(38.4)	87(42.2)	63(34.1)	0.097
Albumin <30g/L	37(9.4)	23(11.2)	14(7.6)	0.225
Prior history of MDRO infection	71(15.5)	32(21.1)	39(18.2)	0.155
Broad-spectrum antibiotic use within the past 3 months	240(61.4)	131(63.6)	109(58.9)	0.343
Neutropenic fever episodes, median(range)	0 (0–7)	0(0-6)	1(0-7)	0.584
Prior hospitalization in other centers	109(27.9%)	55(26.7%)	54(29.2)	0.584
Antibiotic prophylaxis	14(3.6)	7(3.4)	7(3.8)	0.838
Antifungal prophylaxis	145(37.1)	70(34.0%)	75(40.5)	0.180
Invasive procedure during current admission	201(51.4)	106(51.5)	95(51.4)	1.000
Presence of central venous catheter	309(79.0)	162(78.6)	147(79.5)	0.843
≥6 hospital admissions	228(58.3)	122(59.2)	106(57.3)	0.700
ICU transfer during current admission	7(1.8)	2(1.0)	5(2.7)	0.364
Post-HSCT status	12(3.1)	4(1.9)	8(4.3)	0.285
MDRO infection	55(14.1)	32(15.5)	23(12.4)	0.378

AML, Acute Myeloid Leukemia; MDS, Myelodysplastic Syndromes; ECOG, Eastern Cooperative Oncology Group; BMI, Body Mass Index; MDRO, Multidrug-Resistant Organism; ICU, Intensive Care Unit; HSCT, Hematopoietic Stem Cell Transplantation.

**Figure 1:**
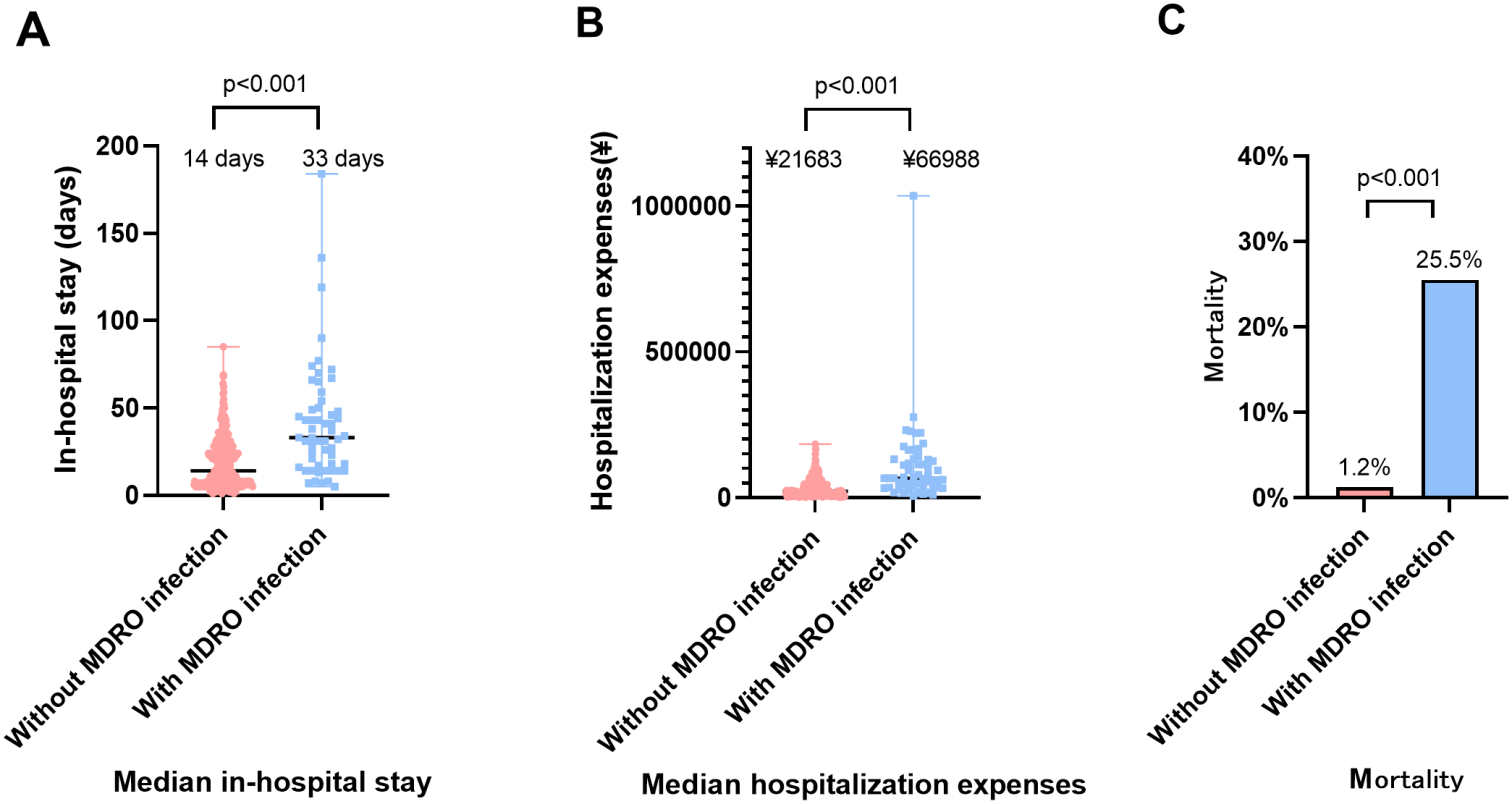
The impact of MDRO infection on clinical outcomes in neutropenic patients. **(A)** Comparison of in-hospital stay between MDRO-infected patients and non-MDRO-infected patients. **(B)** Comparison of hospitalization costs between MDRO-infected patients and non-MDRO-infected patients. **(C)** Comparison of mortality rates between MDRO-infected patients and non-MDRO-infected patients. MDRO, Multidrug-Resistant Organism.

We compared hospitalization duration, medical costs, and mortality rates between MDRO-infected patients and non-MDRO-infected patients in the whole population. The results showed that MDRO-infected patients had significantly longer in-hospital stays (median 33 days vs. 14 days, P<0.001), higher medical expenses (median ¥66988 vs. ¥21683, P<0.001), and higher mortality (25.5% vs. 1.2%, P< 0.001) compared to non-MDRO-infected patients (**Figure 1**).

### 3.2 MDRO infection prediction model variable screening

**Figure 2:**
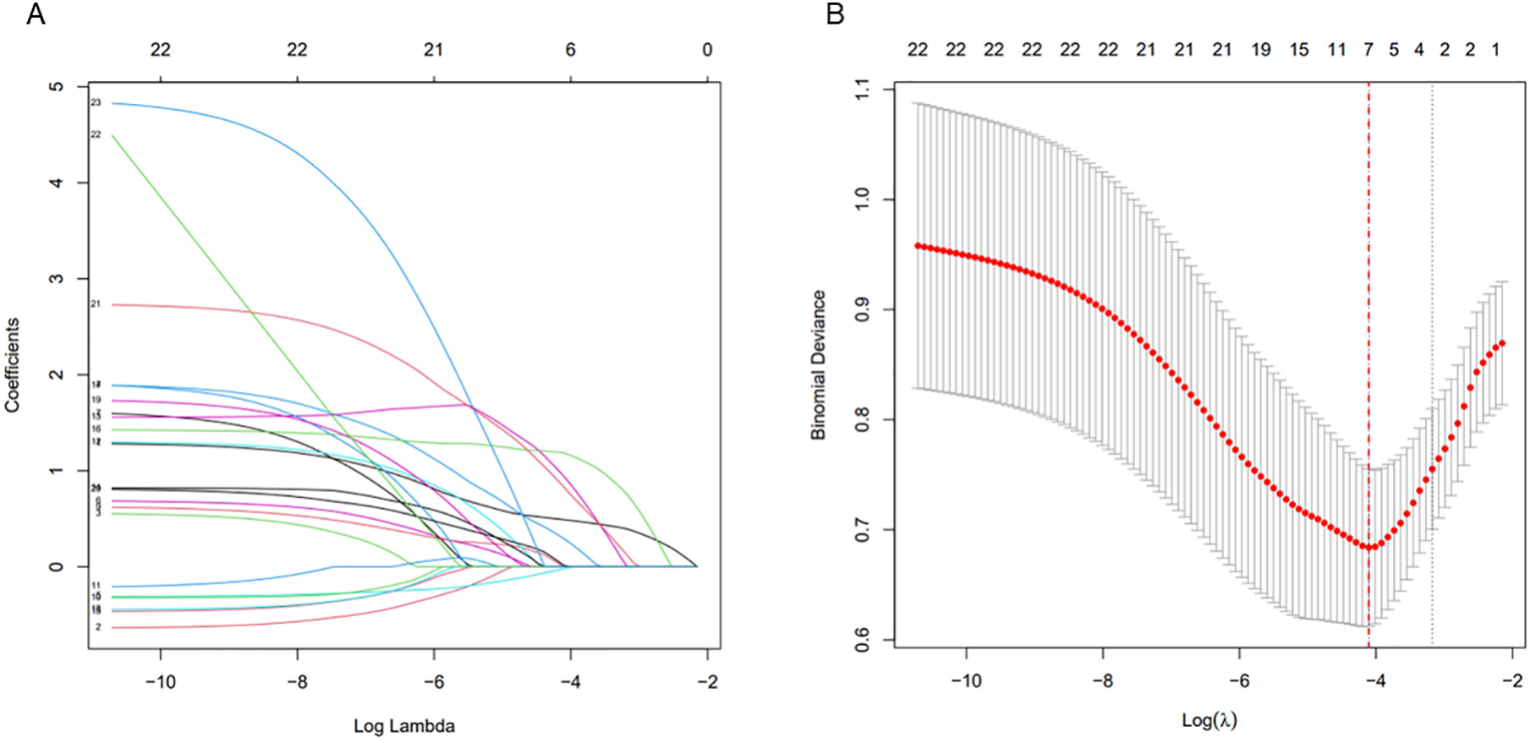
Optimize the screening variables by the LASSO regression. **(A)** Log (lambda) value of 23 variables in the LASSO model. **(B)** Parameter selection in the LASSO model uses ten-fold cross-validation through minimum criterion. LASSO, least absolute shrinkage and selection operator.

In the training cohort, LASSO regression analysis was utilized to identify risk factors. All 23 patient-related characteristics were included in the LASSO regression and 8 variables with non-zero coefficients were identified: comorbid cardiac disease, ECOG score≥2, neutropenia lasting≥7 days, albumin<30 g/L, prior history of MDRO infection, broad-spectrum antibiotic use within the past 3 months, neutropenic fever episodes and prior hospitalization in other centers(**Figures 2A, B**). These 8 variables were then entered into a multivariate logistic regression analysis and finally identified 4 independent risk factors: comorbid cardiac disease (OR = 13.500, 95%CI: 2.484-73.384, P = 0.003), ECOG score≥2 (OR = 3.210, 95%CI: 1.114-9.255, P = 0.031), neutropenia duration≥7 days (OR = 4.028, 95%CI: 1.399-11.600, P = 0.010), and broad-spectrum antibiotic use in 3 months prior (OR = 13.053, 95%CI: 2.419-70.441, P = 0.003) (**Table 2**).

**Table 2:** Multivariate logistic analyses on variables for the prediction of MDRO infection.

Variables	*β*	*SE*	OR (95%CI)	*P* value
Cardiac comorbidity	2.603	0.864	13.500(2.484-73.384)	0.003
ECOG≥2	1.166	0.540	3.210(1.114-9.255)	0.031
Neutropenia lasting ≥7 days	1.393	0.540	4.028(1.399-11.600)	0.010
Albumin <30g/L	1.258	0.961	3.517(0.535-23.108)	0.190
Prior history of MDRO infection	0.890	0.624	2.436(0.717-8.272)	0.154
Broad-spectrum antibiotic use within the past 3 months	2.569	0.860	13.053(2.419-70.441)	0.003
Neutropenic fever episodes	-0.299	0.249	0.742(0.455-1.209)	0.231
Prior hospitalization in other centers	1.012	0.533	2.751(0.968-7.817)	0.058

ECOG, Eastern Cooperative Oncology Group; MDRO, Multidrug-Resistant Organism; OR, odds ratio; CI, confidence interval.

### 3.3 MDRO infection prediction model construction and validation

**Figure 3:**
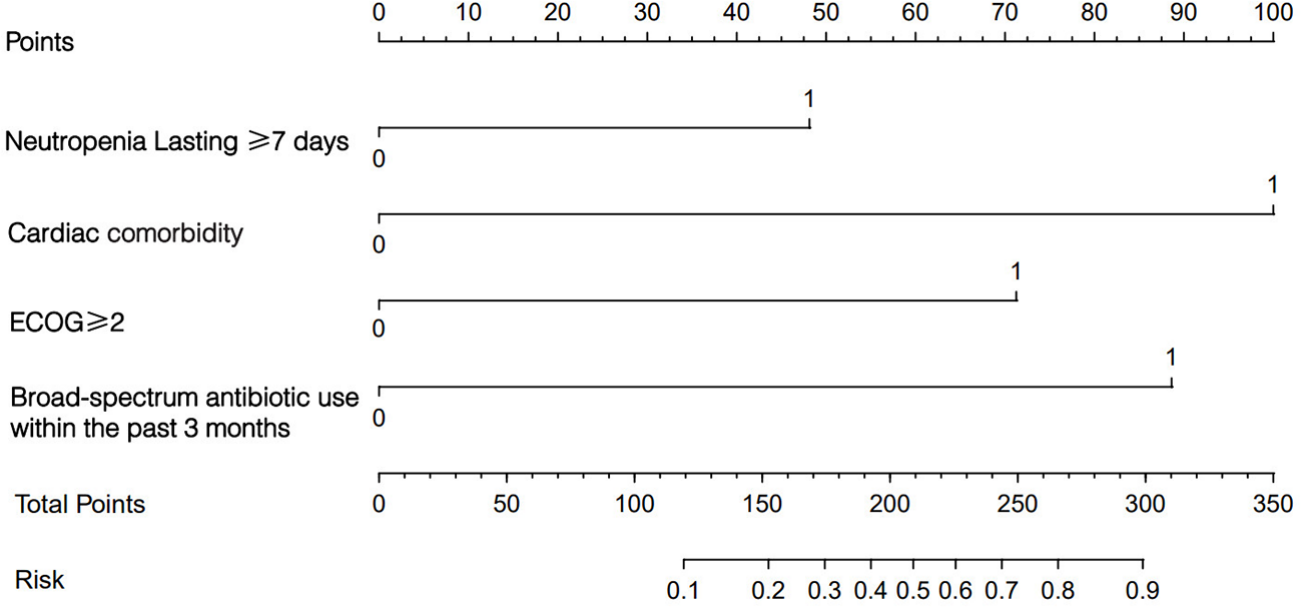
A constructed nomogram of the risk prediction model MDRO infection in neutropenic patients. MDRO, Multidrug-Resistant Organism.

We constructed a nomogram for the prediction of MDRO infection of neutropenic patients according to the variables screened (**Figure 3**). By projecting the total score of the 4 indicators onto the probability line, the risk of MDRO infection can be calculated.

**Figure 4:**
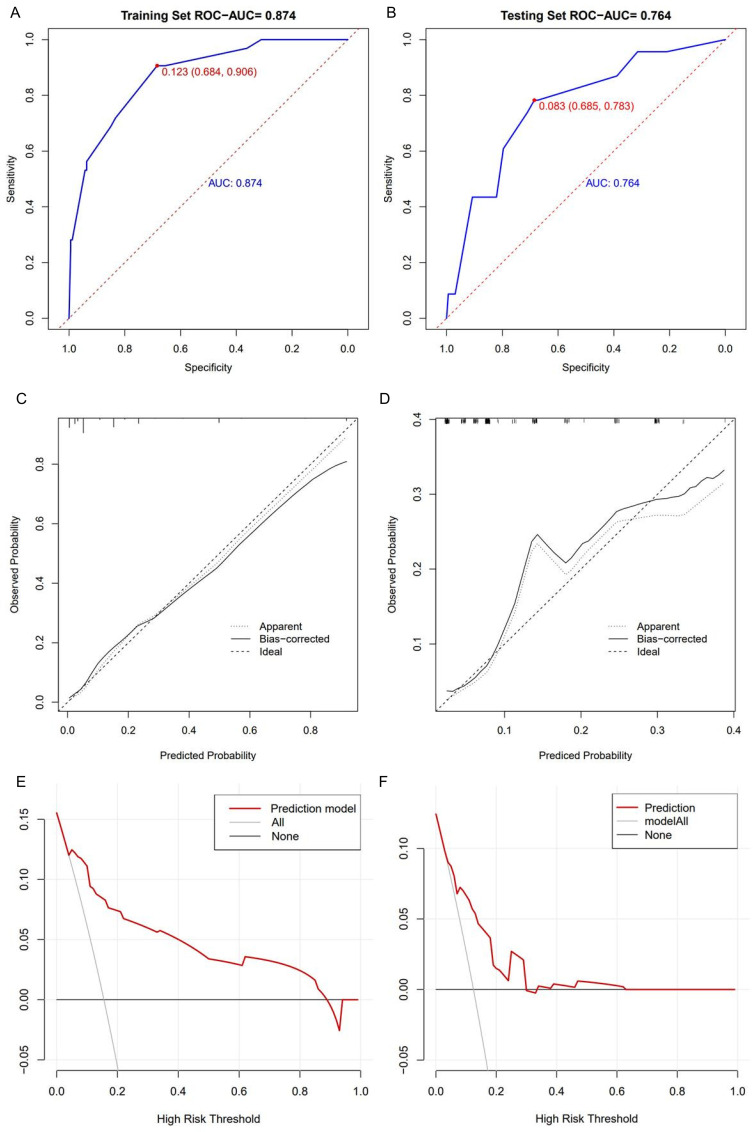
Validation of prediction model. **(A)** ROC curve among the training cohort. **(B)** ROC curve among the validation cohort. **(C)** Calibration curve for the training cohort. **(D)** Calibration curve for the validation cohort. **(E)** DCA of the prediction mode in training cohort. **(F)** DCA of the prediction mode in validation cohort. ROC, receiver operating characteristic; DCA, decision curve analysis.

The ROC curve of the prediction model indicated a sensitivity of 0.684, a specificity of 0.906, and an area under the ROC curve (AUC) of 0.874 (**Figure 4A**). The prediction model was then applied to the validation cohort and showed a model sensitivity of 0.685, a specificity of 0.783, and an AUC of 0.764 (**Figure 4B**). The calibration curves for both the modeling and validation cohorts aligned with the actual curve, indicating that the model had a favorable prediction accuracy (**Figures 4C, D**). The DCA curves showed that the net benefit curve of the nomogram remained consistently above the two reference curves of “intervene in all patients” and “intervene in none” across the majority of the risk threshold range, with an optimal threshold range of 18–30% (**Figures 4E, F**).

## 4 Discussion

The increasing burden of MDRO infections in neutropenic patients has become a critical challenge in hematological clinical practice. The prediction model developed in this study, based on real-world data, provides actionable insights into risk stratification and targeted intervention. The findings of this study indicate that the occurrence of MDRO infections in neutropenic patients is closely associated with multiple independent risk factors, including: Cardiac comorbidity, poor PS (ECOG≥2), neutropenia duration≥7 days and history of broad-spectrum antibiotic use within the past 3 months. These factors collectively contribute to an efficient risk prediction model, with an AUC of 0.874 in the training cohort and 0.764 in the validation cohort, demonstrating good discriminative ability and clinical applicability.

Notably, cardiac comorbidity showed an OR of 13.500 (95% CI: 2.484–73.384), representing a prominent finding. Importantly, cardiac comorbidity was identified as an independent risk factor after adjustment for age, PS status, and other comorbidities in the multivariate model. Although cardiovascular comorbidities are known to increase infection risk in the general population, the markedly heightened susceptibility to MDRO infection in neutropenic patients with such conditions may be mechanistically attributed to the following: Cardiac comorbidities lead to reduced cardiac output and compromised systemic tissue perfusion, thereby impairing immune cell (neutrophils, lymphocytes) trafficking and infiltration to infection sites, while also causing microcirculatory disturbances in the gastrointestinal and respiratory mucosa—disrupting the mucosal barrier and facilitating MDRO colonization and invasion. Concurrently, chronic cardiac dysfunction induces a low-grade systemic inflammatory state that modulates the gut–lung microbiome axis, reduces commensal microbiota diversity, and further promotes the overgrowth of drug-resistant pathogens (Golia et al., 2014; Zhao et al., 2020). These findings suggest that holistic management of cardiac comorbidity, beyond hematological disease control, is integral to MDRO prevention. The use of broad-spectrum antibiotics and the duration of neutropenia, as classic risk factors, align with the European Conference on Infections in Leukemia guidelines proposed by Averbuch et al (Averbuch et al., 2013). Exposure to broad-spectrum antibiotics directly facilitates MDRO colonization by altering gut microbiome diversity, promoting horizontal gene transfer of resistance genes, and eliminating competitive microbiota to create a permissive ecological niche (Bhalodi et al., 2019; Garg et al., 2024). Neutropenia duration≥7 days (OR = 4.028) is associated with delayed bone marrow recovery and prolongs the window for pathogen invasion. ECOG≥2 (OR = 3.210), as a proxy for overall functional status, may imply unquantified confounders such as malnutrition, advanced age, and baseline organ dysfunction.

Previous studies have shown that HSCT is a major risk factor for MDRO bloodstream infections (Girmenia et al., 2015; Forcina et al., 2017). HSCT recipients experience profound and prolonged immunosuppression, compounded by pre-transplant conditioning regimens, graft-versus-host disease, and frequent use of immunosuppressive agents—factors that disrupt the mucosal barrier and microbial homeostasis, creating a permissive environment for MDRO colonization and infection. However, in this study, the sample size of post-HSCT patients may have been too small to detect a significant impact of post-HSCT status on MDRO infection. Future studies with larger sample sizes are needed to further explore this relationship.

The nomogram developed in this study overcomes the limitations of traditional single-indicator prediction, enabling multi-dimensional risk quantification. To our knowledge, this is the first prediction model for MDRO infection in neutropenic patients, with its cross-temporal stability validated in the verification cohort. Furthermore, the DCA further validated its clinical utility. In this study, the median medical costs in the MDRO infection group surged by 3.1 times (¥66,988 vs. ¥21,683), coupled with a 25.5% mortality rate. Each successfully prevented case could save ¥45,000 in direct costs while reducing the risk of death. Therefore, we propose integrating this prediction model into infection prevention and control management plans to identify high-risk MDRO patients early, enabling multimodal and precision infection control interventions (e.g., preemptive microbial screening, empirical antibiotic escalation, enhanced environmental isolation measures, and immunomodulatory interventions (Scheich et al., 2018; Mody et al., 2021; Delanote et al., 2024). This approach aims to reduce the incidence of MDRO infections in neutropenic patients and alleviate the healthcare burden.

This study has several limitations. First, the model was developed and validated in a single hematology center, and the validation cohort was derived from the same hospital in a different time period. The homogeneous patient population and consistent clinical practice patterns in our center may limit the generalizability of the model to other medical centers with different healthcare systems. Application of the model in other centers will require further verification and calibration based on local clinical data. Second, molecular biological markers (such as inflammatory cytokine profiles and resistance gene testing) were not included. Future studies may further optimize this prediction model by incorporating rapid molecular diagnostic assays, such as PCR-based detection of key antibiotic resistance genes. These tools enable early, high-sensitivity identification of MDRO colonization or infection at the preclinical stage, which can be integrated as dynamic variables into the model to enhance real-time risk stratification and early warning capacity. Third, the composite MDRO endpoint may mask subtle pathogen-specific risk patterns due to the heterogeneity of different MDRO subtypes. Given the relatively low incidence of single-pathogen MDRO infection in the current single-center cohort, future multicenter studies with larger sample sizes are warranted to conduct pathogen-specific subgroup analyses and develop subtype-specific prediction models, which will further improve the precision of empirical anti-infective therapy and infection control strategies for neutropenic patients. Fourth, some ORs showed wide 95% CIs due to the low incidence of MDRO infection and low prevalence of certain predictors such as cardiac comorbidity, leading to limited statistical precision. Further external validation in larger multicenter cohorts will help refine estimates and improve precision. Lastly, the dynamic changes in microbial ecology such as the annual increase in MDRO prevalence (Chinese Society of Hematology CMA and Chinese Medical Doctor Association HB, 2020; Li et al., 2020) necessitate regular calibration and updates of the model.

## 5 Conclusions

This study developed a prediction model for MDRO infection in neutropenic patients, identifying four key risk factors: comorbid cardiac disease, ECOG score≥2, neutropenia lasting≥7 days, and broad-spectrum antibiotic use within the past 3 months. More importantly, it established a clinically actionable decision-making tool. By early identification of high risk patients, this enables a paradigm shift from reactive treatment to proactive prevention, ultimately aiming to reduce MDRO infection rates, improve patient survival, and alleviate healthcare burdens.

## Data Availability

The raw data supporting the conclusions of this article will be made available by the authors, without undue reservation.
